# Evaluating the Efficacy of the Erector Spinae Plane Block as a Supplementary Approach to Cardiac Anesthesia during Off-Pump Coronary Bypass Graft Surgery via Median Sternotomy: A Randomized Clinical Trial

**DOI:** 10.3390/jcm13082208

**Published:** 2024-04-11

**Authors:** Sujin Kim, Seung Woo Song, Yeong-Gwan Jeon, Sang A. Song, Soonchang Hong, Ji-Hyoung Park

**Affiliations:** 1 Department of Anesthesiology and Pain Medicine, Wonju College of Medicine, Yonsei University, Wonju 26426, Republic of Korea; sjkim624@yonsei.ac.kr (S.K.); yonfong@yonsei.ac.kr (S.W.S.); ygjeon@yonsei.ac.kr (Y.-G.J.); iad0506@naver.com (S.A.S.); 2 Department of Cardiovascular Surgery, Wonju College of Medicine, Yonsei University, Wonju 26426, Republic of Korea; hongsc93@yonsei.ac.kr

**Keywords:** coronary artery disease, cardiac surgical procedures, sternotomy, myocardial revascularization, erector spinae plane block, pain, postoperative

## Abstract

**Background:** Pain control after off-pump coronary artery bypass graft (OPCAB) facilitates mobilization and improves outcomes. The efficacy of the erector spinae plane block (ESPB) after cardiac surgery remains controversial. **Methods:** We aimed to investigate the analgesic effects of ESPB after OPCAB. Precisely 56 patients receiving OPCAB were randomly divided into ESPB and control groups. The primary outcome was visual analog scale (VAS) pain scores at 6, 12, 24, and 48 h postoperatively. Secondary outcomes were the dose of rescue analgesics in terms of oral morphine milligram equivalents, the dose of antiemetics, the length of intubation time, and the length of stay in the intensive care unit (ICU). **Results:** The VAS scores were similar at all time points in both groups. The incidence of severe pain (VAS score > 7) was significantly lower in the ESPB group (50% vs. 15.4%; *p* = 0.008). The dose of rescue analgesics was also lower in the ESPB group (19.04 ± 18.76, 9.83 ± 12.84, *p* = 0.044) compared with the control group. The other secondary outcomes did not differ significantly between the two groups. **Conclusions:** ESPB provides analgesic efficacy by reducing the incidence of severe pain and opioid use after OPCAB.

## 1. Introduction

Despite the advantages of reduced transfusion and hospital stay, off-pump coronary artery bypass grafting (OPCAB) is not preferred over on-pump coronary artery bypass grafting (CABG) because of the risk of ineffective revascularization [1]. However, contrary to these results, there are studies indicating that both CABG and OPCAB are safe options, and OPCAB is still being performed in many cases [2]. Although OPCAB does not use a cardiopulmonary bypass machine, it uses a sternal retractor, internal mammary artery harvester, and chest tube after surgery [3]. Persistent chest pain is related to the intensity of acute pain after surgery, and 22% of patients continue to experience pain 3 months after sternotomy [4]. Therefore, similar to other cardiac surgeries, pain control after OPCAB is a key factor for facilitating patient mobilization and improving recovery.

Since its introduction in 2016, the erector spinae plane block (ESPB) has been gaining popularity in various surgeries owing to its ease of implementation and low risk of adverse effects [5]. However, evidence remains insufficient to confirm the efficacy of various block techniques performed after cardiac surgery, and there is no gold standard for pain management after cardiac surgery. 

This trial aimed to investigate the efficacy of ESPB after OPCAB. We hypothesize that ESPB reduces pain scores after surgery and the dose of rescue analgesics used (calculated as the oral morphine milligram equivalent, [MME]). 

## 2. Materials and Methods

### 2.1. Study Design

This was a single-center, double-blind, randomized controlled trial. This study was approved by the Institutional Review Board of Yonsei University in Wonju and ensured compliance with ethical standards, patient confidentiality, and adherence to relevant regulatory guidelines (CR322089). As part of ethical practice and transparency in clinical research, all participants involved in this study were appropriately registered in Korea’s Clinical Research Information Service, ensuring their inclusion and documentation in a national database (https://cris.nih.go.kr/, accessed at KCT0008781; registration date: 30 August 2023). 

### 2.2. Study Population

We enrolled 56 patients aged 19 years or older with a diagnosis of coronary artery disease scheduled for OPCAB surgery at Severance Christian Hospital in Wonju, Korea, from December 2022 to August 2023. All the patients underwent surgery performed by one cardiovascular surgeon (S.H.). We excluded patients with contraindications to nerve block surgery (coagulopathy, infection at the procedure site, allergic reaction to local anesthetic, or refusal to undergo the procedure) from the study. Patients with comorbidities (sepsis, thoracic deformity, and increased intracranial pressure) who were excluded at the discretion of the anesthesiologist, those who had difficulty giving voluntary consent due to illiteracy or cognitive dysfunction, and those who underwent emergency surgery were also excluded from this study. 

### 2.3. Randomization and Blinding

The patients were randomly assigned into ESPB and control groups using a computer. Except for one researcher who performed the ESPB (S.K.), the patients and other physicians participating in this study did not know to which group the patients were assigned. The assignments were secured in sequentially numbered sealed opaque envelopes. At the end of the surgery, one of the authors opened the envelope containing the study assignment and performed a pain block procedure. After the patient was transferred to the intensive care unit (ICU), the patient and researcher who participated in data collection (S.S.) were blinded.

### 2.4. Perioperative Management

Midazolam (0.05 mg/kg), sufentanil (1–2 mcg/kg), rocuronium (0.8 mg/kg), and sevoflurane were used to induce anesthesia. After tracheal intubation, Arrowg+ard Blu^®^ MAC (Teleflex Inc., Limerick, Ireland) and Swan-Ganz^®^ (Edwards Lifesciences, Irvine, CA, USA) catheters were inserted to facilitate intraoperative monitoring for right heart function (e.g., pulmonary artery pressure, central venous pressure, cardiac index of right heart, etc.). Transesophageal echocardiography was performed to monitor cardiac contractility, lusitropy, and wall motion abnormalities during surgery. Anesthesia was maintained via the inhalation of sevoflurane and infusion of sufentanil and rocuronium. The sevoflurane inhalation rate was controlled using Sedline electroencephalography guidance (Masimo, Irvine, CA, USA). The sufentanil infusion rate was adjusted according to the overall hemodynamic data by the attending anesthesiologist, who suggested that the intensity of the surgical stimuli and rocuronium be adjusted by a train of four watches within the surgical neuromuscular block range. At the end of the surgery, the patient was placed in the lateral decubitus position for ESPB. One of the authors (S.K.) performed ESPB under ultrasonographic guidance to ensure the accuracy and efficacy of the block. Subsequently, as part of the postoperative pain management strategy, an intravenous patient-controlled analgesia (PCA) regimen was prescribed by the attending anesthesiologist. This PCA system was designed to deliver analgesia tailored to individual patient needs and contained an approximate dosage of 18 mcg/kg of fentanyl, allowing for controlled and adjustable pain relief in response to patient-initiated dosing. The patients were transferred to the ICU, and the primary physician (S.H.) oversaw postoperative pain management. The dosage of postoperative analgesics was recorded in MME. Intravenous tramadol (50 mg), intramuscular or subcutaneous meperidine (25 mg), oral Ultracet^®^ (Janssen Korea Ltd., Seoul, Republic of Korea; tramadol [37.5 mg]/acetaminophen [325 mg]), and transdermal fentanyl patches (25 mcg/h) were used postoperatively. Withdrawal from the study was considered in cases of conversion to on-pump surgery or hypersensitivity reactions during anesthetic induction. 

### 2.5. Erector Spinae Plane Block

After the surgical procedure, the patient was placed in the lateral decubitus position for the ESPB. The skin overlying the intended block area was prepared and disinfected with betadine. Using ultrasound guidance, the anatomical structures were visualized to confirm the T5 level and relevant surrounding structures. The trapezius, rhomboid, and erector spinae muscles were visualized via an ultrasound to guide the insertion of an echogenic needle (Echoplex^®^; Vygon, France) into the desired plane. Using an in-plane ultrasound approach, the needle was advanced until it reached the plane of the erector spinae muscles. The needle position was confirmed by hydrodissection using 2–3 mL of saline. Once the correct placement of the needle within the erector spinae plane was confirmed, a mixture of local anesthetics was injected. The administered dose was a 20 mL mixture consisting of 0.225% ropivacaine, 1% lidocaine, and normal saline. The ESPB was performed with a total volume of 40 mL, with 20 mL on each side. 

### 2.6. Outcome Measures

The primary outcome of this study was the assessment of postoperative pain intensity using the Visual Analog Scale (VAS) at 6, 12, 24, and 48 h postoperatively. The VAS, ranging from 0 (no pain) to 10 (worst imaginable pain), provided a quantitative measurement of the pain experienced by the patients. The highest VAS score was recorded at each time point. 

Secondary outcomes measured included the dose of rescue analgesics injected into patients (in MME), the dose of antiemetics used, the length of intubation time, and the length of stay in the ICU. 

### 2.7. Sample Size Calculation

The sample size calculation was derived from a previous study that reported a mean postoperative pain score of 5.48 ± 1.09 when utilizing opioid-based intravenous PCA following cardiac surgery [6]. Considering a clinically meaningful reduction of more than 1 point in the average pain score, with a type I error of 0.05 and a power of 0.90, a sample size of 25 individuals per group was required. Factoring in a potential dropout rate of 10%, the study aimed to include 56 participants, with 28 individuals allocated to each group.

### 2.8. Statistical Analysis

All statistical analyses were performed using the IBM SPSS statistical software (IBM SPSS Statistics for Windows, version 27, IBM Corporation, Armonk, NY, USA). Continuous variables were presented as the mean ± standard deviation (SD). For continuous variables such as postoperative pain intensity (measured by VAS) and time from the end of anesthesia to extubation, a comparison between the intervention and control groups was conducted using the independent samples *t*-test. This statistical test was used to assess whether there were significant differences in the means of the continuous variables between the two groups. The significance level was set at *p* < 0.05, and 95% confidence intervals were calculated to estimate the precision of the mean differences between the groups. The Mann–Whitney U test was performed to ensure the accuracy of the analysis. Regarding categorical variables, such as the presence or absence of perioperative complications, relevant statistical tests, such as the chi-square test, were used to evaluate the association or difference in frequency between the intervention and control groups. Repeated measurements of pain scores were corrected using post hoc Bonferroni correction.

## 3. Results

**Figure 1:**
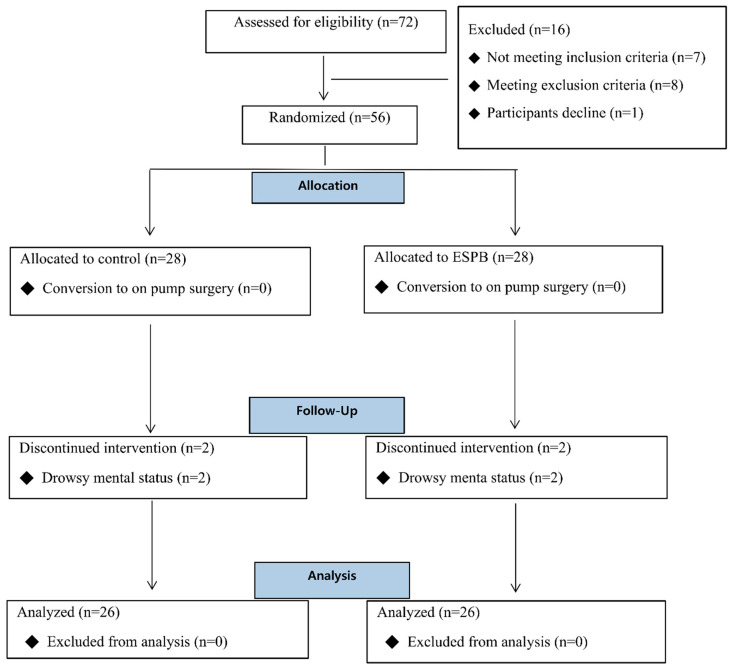
CONSORT flow chart.

Initially, 72 patients were assessed for eligibility, and 16 patients were excluded for the following reasons, including surgery performed on-pump CABG (*n* = 7) and emergency surgery (*n* = 8), and the participants declined (*n* = 1). A total of 56 patients enrolled in this study were assigned to the intervention and control groups; 4 patients dropped out because of drowsy mental status (Figure 1). Patients’ characteristics and surgery data are shown in Table 1.

There were no statistically significant differences in the VAS scores at 6, 12, 24, and 48 h after surgery between the two groups. Although there was no significance after repeated measurement correction, pain tended to be lower at 6 h (4.46 ± 1.98 vs. 5.96 ± 1.84, *p* = 0.020). However, the incidence of severe pain was significantly lower in the ESPB group than in the control group (15.4% vs. 50%, *p* = 0.008). Additionally, the dose of rescue analgesics was significantly reduced in the ESPB group (19.04 ± 0.49 vs. 9.83 ± 12.84, *p* = 0.044). The lengths of ICU and hospital stays were similar. The time to extubation did not differ between the two groups (Table 2).

In the multiple regression analysis of VAS at 6 h, taking into account the influence of modifying factors, only ESPB was significantly related to the pain score (Table 3).

**Figure 2:**
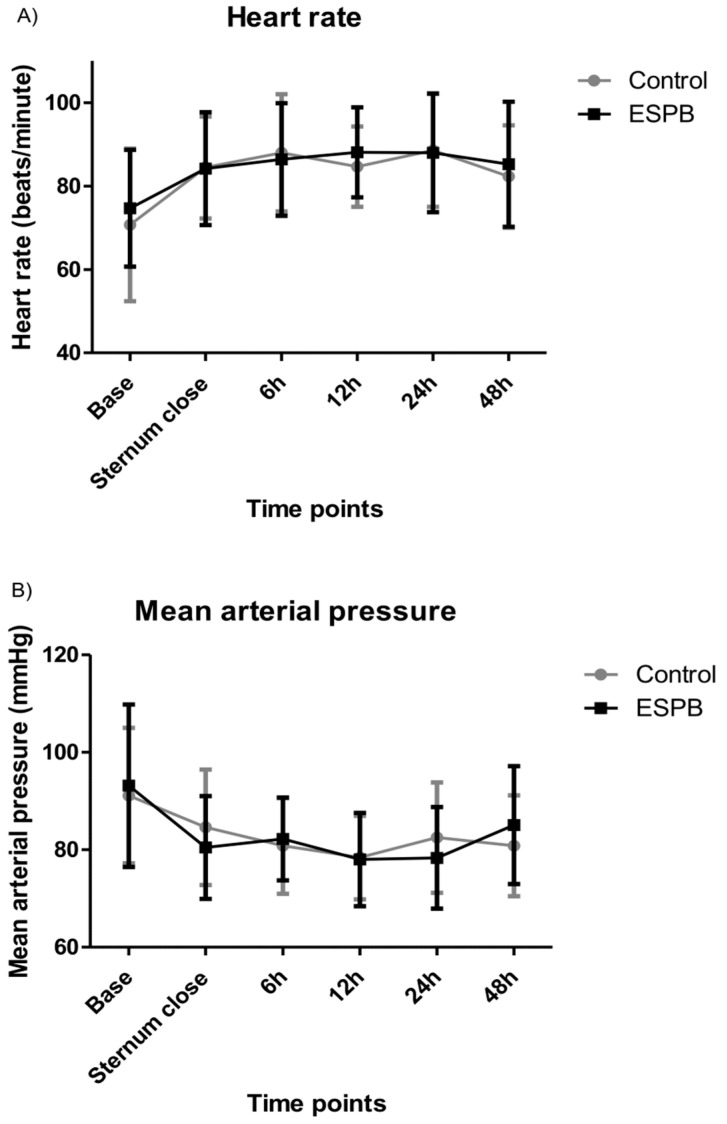
Hemodynamic data: (**A**) heart rate; (**B**) mean arterial pressure. Hemodynamic data did not significantly differ between the ESPB and control group.

The heart rate and mean arterial pressure (MAP) were similar between the two groups (Figure 2). 

## 4. Discussion

### 4.1. Discussion

This trial demonstrated that ESPB did not reduce pain scores, which was the primary outcome. However, the incidence of severe pain (VAS score, 7–10) and the dose of rescue analgesics were significantly reduced compared to those in the control group. Although the ESPB did not reduce the pain score, the VAS score at six hours postoperatively showed a statistically significant correlation with ESPB, considering the modifying factors. 

In a previous prospective cohort study on 213 patients who underwent CABG surgery, 78% of patients experienced severe pain during coughing, and 49% experienced severe pain at rest [7]. Acute postoperative pain reduces pulmonary function parameters such as tidal volume, vital capacity, functional residual capacity, and pulmonary compliance [8]. The continuous stimulation of the sympathetic nervous system causes an oxygen supply-demand mismatch in the myocardium and dysfunction of the gastrointestinal and urinary systems. Prolonged nociceptor stimulation suppresses the immune system, leading to the infection or inhibition of wound healing [9]. Additionally, severe immediate postoperative pain is associated with the incidence of chronic pain [10]. 

Despite the cardioprotective function of opioids, it is recommended that their use is limited to anesthetic practice using a multimodal approach. Opioid-induced hyperalgesia and chronic neuropathic pain require another approach to replace opioids from a patient-centered care perspective after cardiac surgery [11]. Additionally, 21.7% of patients continue to use opioids for 3 months after CABG, and the global crisis of opioid dependency has become a major healthcare issue [12]. Various regional block techniques have been investigated; however, evidence supporting their use as standard treatments is lacking [13]. ESPB is a myofascial block similar to the effect of local anesthetics but spreads to the paravertebral and epidural spaces, affecting the dorsal and ventral rami of the spinal nerves [5]. Although its exact mechanism is not well understood, it is a clinical option widely used in various surgeries [14]. OPCAB does not use a cardiopulmonary bypass, and systemic inflammation is less than that in CABG; therefore, the intensity and incidence of pain are lower [15]. Pain after OPCAB occurs because of prolonged sternal retraction and the presence of chest tubes. Rib fractures, costochondritis, rib joint dislocation, left internal mammary artery harvesting, or nerve injury may occur due to sternal retraction. The presence of a chest tube may irritate the parietal pleura or pericardium [16]. The sternum is mainly innervated by spinal nerves T2–T6; however, pain is perceived in a wide area beyond the dermatome because of secondary tissue injury from distant sites [16]. In a previous systematic review, a thoracic epidural block (TEB) reduced the risk of mortality, and the number of patients treated for hematoma was 1:3552, which is very rare [17]. Nevertheless, because of the severity of the complications, there are limited works in the literature guaranteeing the efficacy of TEB in cardiac surgeries, even in OPCAB surgery, which maintains a relatively low anticoagulation time. Therefore, more superficial techniques with a lower risk of complications should be considered [18]. 

Our results indicated that patients who underwent ESPB experienced less severe pain despite using a lower dose of rescue analgesics after OPCAB surgery. Lower pain scores were observed in the ESPB group than in the control. This beneficial effect is in line with a previous study showing that ESPB reduced pain scores and rescue analgesics after cardiac surgery using CABG [19]. In a previous prospective observational study that investigated the intensity and location after cardiac surgery, the most painful area was the incisional site, and pain intensity diminished from postoperative day 2 [20]. Therefore, controlling acute pain by targeting the intercostal nerve distributed to the sternum is key to analgesia after OPCAB surgery. When performing ESPB, the spread of local anesthetics into the paravertebral space acting on the intercostal nerve, as well as its craniocaudal spread, may be responsible for pain reduction [5]. 

Regarding the reduction in rescue opioid use, we did not observe significant additional benefits such as shorter extubation time, length of stay, or reduction in the dose of antiemetics. The rescue analgesic doses (19.04 ± 0.49 vs. 9.83 ± 12.84, *p* = 0.044) used in this study were similar to a previous study (19.0 ± 2.6 vs. 11.2 ± 1.6, *p* < 0.001) comparing ESPB to intravenous PCA after CABG [21]. Despite this significant difference, the rescue doses of opioids used in both groups were too small to confirm the additional benefits. 

Adverse effects related to ESPB were similar between the two groups. No significant differences were observed in the incidences of hypotension, bradycardia, or pneumothorax. In a previous review, ESPB-related complications were rare, and the procedure could be safely performed [14].

### 4.2. Strengths and Limitations

Since it was first described in 2016, publications relating to ESPB have increased remarkably, more than any other block technique [22]. However, there are only a limited number of RCTs [23]. To the best of the authors’ knowledge, there are two studies evaluating the effectiveness of ESPB in patients undergoing OPCAB [24,25]. Both studies showed that ESPB was effective. However, one study was retrospective [24], and the other was an observational study [25]. The current trial is a high-quality RCT; therefore, it provides primary evidence that performing ESPB in OPCAB patients may be a safe analgesic option for the primary care physician. In addition, the greatest strength of ESPB is the ease of implementation. In a previous study, the success rate of ESPB and the paravertebral block performed by residents was significantly higher in the ESPB group (100% vs. 77.8%, *p* = 0.002), and the duration to a performed block was short (4.39 ± 1.20 min vs. 8.18 ± 2.42 min, *p* < 0.001) [26]. Under ultrasound guidance, the transverse process can be easily distinguished and distanced from prominent structures, such as the pleura and vessels, as a skill that could be easily acquired. 

This trial had several limitations. First, we used a large amount (18 mcg/kg of fentanyl) of opioid-based PCA in both groups. This may be a confounding factor in guaranteeing the block’s efficacy. An opioid-based analgesic regimen has been used and adjusted at our institution over the past decade to provide acceptable pain relief. Nevertheless, additional ESPB reduces the need for rescue analgesics, and the incidence of severe pain would be meaningful as a multimodal approach. Second, we only assessed pain scores for up to 48 h. The effectiveness of ESPB would have been more certain if we had evaluated the incidence of chronic pain or changes in the location and intensity of pain over time. However, we already know that pain decreases after 3 days [20] and that the severity of acute pain is a major factor in the transition to chronic pain. Therefore, pain for up to 48 h may be the most important assessment criterion. Third, the sample size may be insufficient for validating the incidence of severe pain, and the dose of analgesic rescues and clinical application may be limited because of the small number of participants. Fourth, we assessed the efficacy of ESPB in a single shot. If we used a continuous block with a catheter or a longer-acting local anesthetics, the analgesic efficacy of ESPB would have been better exhibited. Finally, we performed ESPB with the patient sedated; therefore, block failure might not have been recognized. 

### 4.3. Conclusions

In conclusion, OPCAB requires proper analgesic pain relief without adverse effects such as hematoma or pneumothorax. In line with these needs, the present trial demonstrates that ESPB lowers the incidence of severe pain and reduces the dose of opioids required. 

## Figures and Tables

**Table 1:** Patients’ characteristics and surgery data.

	Control Group (*n* = 26)	ESPB Group (*n* = 26)	*p* Value
Age (years)	64.58 ± 11.07 [43, 83]	63.65 ± 10.83 [47, 85]	0.602
Female	6 (23.1)	4 (15.4)	0.482
Height (cm)	163.75 ± 9.30 [144.0, 179.1]	164.79 ± 7.97 [145.0, 183.0]	0.667
Weight (kg)	66.82 ± 13.63 [44.42, 94.40]	70.33 ± 11.11 [49.42, 90.10]	0.314
Hypertension	20 (76.9)	21 (80.8)	0.734
Diabetes mellitus	16 (61.5)	16 (61.5)	1.000
Cerebral vascular disease	5 (19.2)	2 (7.7)	0.223
Chronic kidney disease	4 (15.4)	6 (23.1)	0.482
NYHA class	2.19 ± 0.98 [1, 4]	2.00 ± 0.80 [1, 4]	0.442
CCS score	2.19 ± 0.98 [1, 4]	2.04 ± 0.72 [1, 4]	0.522
EuroSCORE	1.73 ± 1.50 [0.50, 6.29]	1.45 ± 0.73 [0.50, 3.01]	0.397
Heart failure	10 (38.5)	10 (38.5)	1.000
Ejection fraction (%)	49.19 ± 12.67 [26, 67]	49.73 ± 11.54 [27, 72]	0.873
Surgery time (min)	243.31 ± 33.27 [175, 325]	239.54 ± 31.14 [171, 315]	0.675
Anesthesia time (min)	327.31 ± 35.92 [270, 420]	331.73 ± 33.67 [250, 420]	0.649

Values are displayed as the mean ± SD [minimum, maximum] or n (%). NYHA, New York Heart Association; CCS, Canadian Cardiovascular Society.

**Table 2:** Outcome comparisons between the groups.

	Control Group (*n* = 26)	ESPB Group (*n* = 26)	*p* Value	Mean Difference (95% CI)
Primary Outcomes				
VAS 6 h	5.96 ± 1.84 [2, 9]	4.46 ± 1.98 [0, 9]	0.020 *	−1.27 (−2.33, −0.20)
VAS 12 h	4.58 ± 2.25 [2, 9]	3.85 ± 1.87 [0, 9]	0.208	−0.73 (−1.88, 0.42)
VAS 24 h	3.73 ± 2.07 [1, 9]	3.62 ± 1.75 [1, 8]	0.829	−0.12 (−1.18, 0.95)
VAS 48 h	2.85 ± 2.39 [0, 10]	2.00 ± 1.62 [0, 6]	0.143	−0.85 (−1.99, 0.29)
VAS (7–10)	13 (50)	4 (15.4)	0.008 *	
Secondary outcomes				
Number of rescue analgesic events	1.27 ± 1.25 [0, 4]	0.73 ± 0.92 [0, 3]	0.083	−0.54 (−1.15, 0.07)
Rescue analgesic dose (MME)	19.04 ± 18.76 [0, 60.0]	9.83 ± 12.84 [0, 45.0]	0.044 *	−9.21 (−18.16, −0.25)
Number of antiemetic agents required	0.19 ± 0.49 [0, 2]	0.15 ± 0.54 [0, 2]	0.790	−0.04 (−0.33, 0.25)
New onset atrial fibrillation, *n* (%)	3 (11.5)	3 (11.5)	1.000	
Pneumothorax	0 (0)	0 (0)	1.000	
Wound infection	1 (3.8)	0 (0)	0.313	
Mortality	0 (0)	0 (0)	1.000	
ICU duration (day)	1.27 ± 0.83 [1, 4]	1.12 ± 0.33 [1, 2]	0.382	−0.15 (−0.50, 0.20)
Hospital day (day)	12.50 ± 12.50 [6, 68]	9.73 ± 2.41 [7, 16]	0.277	−2.78 (−7.89, 2.36)-
Time to extubation (min)	671.15 ± 528.07 [185, 2835]	561.15 ± 263.26 [185, 1125]	0.346	−110.00 (−342.43, 122.43)

Values are displayed as the mean ± SD [minimum, maximum] or n (%). The pain score was assessed using a VAS (0 = no pain, 10 = worst imaginable pain). The rescue analgesic requirement was calculated in MME. VAS: visual analog scale; MME: morphine milligram equivalent; ICU: intensive care unit. SD: standard deviation, CI: confidence interval, * = *p* value < 0.05.

**Table 3:** Multiple regression analysis of VAS at 6 h.

Variable	Regression Coefficient	T Value	*p* Value
ESPB	−1.386	−2.331	0.025 *
Sex	−0.602	−0.754	0.455
Age	0.001	0.028	0.978
DM	−0.117	−0.182	0.978
EuroSCORE	−0.362	−0.182	0.856
Operation time	−0.008	−1.187	0.242
Graft number	0.084	0.170	0.866
Ejection fraction	0.021	0.712	0.481
Dose of Norepinephrine	−0.001	−0.096	0.924

ESPB is significantly related with VAS pain score at 6 h postoperatively. * = *p* value < 0.05.

## Data Availability

The data presented in this study are available upon request from the corresponding author.
